# Automated extraction of fluoropyrimidine treatment and treatment-related toxicities from clinical notes using natural language processing

**DOI:** 10.1016/j.ijmedinf.2026.106276

**Published:** 2026-01-10

**Authors:** Xizhi Wu, Madeline S. Kreider, Philip E. Empey, Chenyu Li, Yanshan Wang

**Affiliations:** a Department of Health Information Management, University of Pittsburgh, Pittsburgh, PA, USA; b Department of Pharmacy & Therapeutics, University of Pittsburgh, Pittsburgh, PA, USA; c Department of Biomedical Informatics, University of Pittsburgh, Pittsburgh, PA, USA; d Intelligent Systems Program, University of Pittsburgh, Pittsburgh, PA, USA; e Clinical and Translational Science Institute, University of Pittsburgh, Pittsburgh, PA, USA

**Keywords:** Fluoropyrimidine, Large language models, Natural language processing, Clinical notes, Toxicities, Electronic Health Record

## Abstract

**Objective:**

Fluoropyrimidines are widely prescribed for colorectal and breast cancers, but are associated with toxicities such as hand-foot syndrome and cardiotoxicity. Since toxicity documentation is often embedded in clinical notes, we aimed to develop and evaluate natural language processing (NLP) methods to extract treatment and toxicity information.

**Materials and methods:**

We constructed a gold-standard dataset of 236 clinical notes from 204,165 adult oncology patients. Domain experts annotated categories related to treatment regimens and toxicities. We developed rule-based, machine learning-based (Random Forest [RF], Support Vector Machine [SVM], Logistic Regression [LR]), deep learning-based (BERT, ClinicalBERT), and large language models (LLM)-based NLP approaches (zero-shot and error analysis prompting). A 5-fold cross validation were conducted to validate each model.

**Results:**

Error analysis prompting achieved optimal precision, recall, and F1 scores for treatment (F1 = 1.000) and toxicities extraction (F1 = 0.965), whereas zero-shot perform moderately (treatment F1 = 0.889, toxicities extraction F1 = 0.854) Rule-based reached F1 = 1.000 for treatment and F1 = 0.904 for toxicities extraction. LR and SVM ranked second and fourth for toxicities extraction (LR F1 = 0.914, SVM F1 = 0.903). Deep learning and RF underperformed, with performance of BERT reached F1 = 0.792 for treatment and F1 = 0.837 for toxicities extraction.,ClinicalBERT reached F1 = 0.797 for treatment and F1 = 0.884 for toxicities extraction). RF reached F1 = 0.745 for treatment and F1 = 0.853 for toxicities extraction.

**Discussion:**

LMM-based error analysis outperformed all others, followed by machine learning methods. Machine learning and deep learning methods were limited by small training data and showed limited generalizability, particularly for rare categories.

**Conclusion:**

LLM-based error analysis most effectively extracted fluoropyrimidine treatment and toxicity information from clinical notes, and has strong potential to support oncology research and pharmacovigilance.

## 1. Background and significance

Fluoropyrimidines (FPs), including capecitabine and 5-fluorouracil (5-FU), are chemotherapy agents often used in the treatment of colorectal and breast cancers [1]. However, FP use is associated with multiple adverse events, including hand-foot syndrome (HFS), cardiotoxicity and gastrointestinal toxicities [2]. One of the most common adverse drug reactions (ADRs) for patients taking capecitabine is HFS, occurring in up to 42 % of breast cancer patients and 53.5 % of colorectal cancer patients [1], and a median onset of 79 days [2]. HFS is a dermatologic reaction affecting the skin of the palms of the hands and soles of the feet. HFS presents as painful redness, swelling, tingling, and in more severe cases blistering, peeling, or thickening of the skin [3]. Cardiotoxicity is a serious but rare toxic ADR [2] that occurs in approximately 5 % of patients receiving a FP [4,5]. It presents with chest pain, palpitations, dyspnoea, hypotension, arrhythmias, and in rare cases may lead to myocardial infarction, heart failure, or sudden cardiac death [5]. These toxicities can significantly impair patients’ quality of life, clinical outcomes, and treatment course, often leading to medication interruptions, discontinuations, and dose or frequency decreases [6,7]. Large-scale and accurate identification of these toxicities in electronic health records (EHRs) is critical for advancing clinical research and ultimately informing strategies for better toxicity prediction, prevention, and treatment.

EHRs are valuable sources to find and extract FP treatment and treatment-related toxicity information. ADRs are often documented in unstructured clinical notes rather than structured fields [8]. There are 3 commonly used methods in extracting such data from EHRs: International Classification of Diseases (ICD) codes from structured EHRs, manual chart review of free-text clinical notes, and natural language processing (NLP) of free-text clinical notes.

Manual chart review is a retrospective examination of individual patient medical records by trained reviewers to identify FP toxicities. This method allows researchers to capture ADRs, dose modifications, or reasons for treatment discontinuation [9–11], as documented in physician notes or other unstructured sections of the EHR. Many studies have relied on manual review of free-text notes to extract FP toxicity information. Jiang et al. [12] assess how older patients tolerate capecitabine by having two trained annotators analyzing toxicities such as HFS, fatigue and diarrhea from EHRs and patient reported outcomes (PRO). Hennessy et al. [9] conducted a manual review of oncology patients’ records at M.D. Anderson Cancer Center to extract detailed toxicity data associated with different starting doses of capecitabine. Van Beek et al. [13] conducted a retrospective review of EHRs from the oncology department of Radboud University Medical Center. Capecitabine toxicities were manually identified and graded using the NCI CTCAE v3.0 (Common Terminology Criteria for Adverse Events) [14]. While manual chart review provides high clinical fidelity, it is time-consuming [15], resource-intensive, and subject to documentation variability and inter-rater inconsistency [16]. Therefore, we propose NLP methods to reduce the effort and time needed for identifying FP treatment and treatment-related toxicities from unstructured clinical text.

Structured diagnosis codes, such as the International Classification of Diseases (ICD), are commonly used in claims and administrative data to identify ADRs. For example, Nishijima et al. [17] utilized ICD-9 codes from Medicare records to extract ADRs including diarrhea, dehydration, and cardiotoxicity in older adults treated with capecitabine. Bhimani et al. [17] extracted capecitabine-related toxicities from EHR-derived structured data using predefined ICD-9/10 diagnosis codes, laboratory values, and treatment timelines. Li et al. [18] identified cardiotoxicity events using predefined ICD-9-CM diagnosis codes for cardiac conditions such as heart failure, arrhythmia, and myocardial infarction, treating these events as indicators of chemotherapy-induced cardiotoxicity. However, reliance on billing codes may lead to underreporting or misclassification [19,20], particularly for lower-grade or undocumented toxicities that are not formally coded. While ICD-based extraction provides scalability and standardization, it lacks the detail available from the clinical notes within EHR to appropriately identify patients with and without the toxicity [21]. Our study focuses on extracting FP Treatment and treatment-related toxicities from unstructured EHR narratives. Future work may involve integrating NLP-based approach with structured data sources, such as ICD-coded diagnoses, to improve coverage and accuracy.

Natural language processing (NLP) is a technique widely used to extract ADRs from unstructured EHR data [22]. NLP is a field of artificial intelligence focused on enabling computers to understand, interpret, and generate human language [23]. In the clinical domain, NLP techniques are applied to extract meaningful information from unstructured text, such as physician notes, discharge summaries, and progress reports [24]. These texts often contain rich clinical narratives, including detailed descriptions of symptoms, adverse events, and clinical decisions that are not recorded in objective, structured data fields. In terms of extracting ADRs, NLP methods include a range of machine learning techniques, including neural sequence labeling models like bidirectional Long Short-Term Memory (LSTM) networks with a Conditional Random Field (CRF) layer (BiLSTM-CRF) [25] for named entity recognition (NER) and models such as attention-based LSTM [26], Support Vector Machine (SVM) [27], and Random Forest (RF) [28] for relation classification [29]. Hong et al. [30] developed an NLP pipeline based on Apache Mayo clinical Text Analysis and Knowledge Extraction System (cTAKES) [31] to automatically extract Common Terminology Criteria for Adverse Events (CTCAE) [32] v5.0-defined toxicities related to capecitabine and other chemotherapies. The NLP pipeline extracted from free-text oncology treatment notes using Unified Medical Language System (UMLS)-based NER, assertion detection, and post-processing rules. The NLP pipeline by Hong was validated against expert annotation and achieved a precision of 0.91, recall of 0.72, and F1 score of 0.80 in extracting CTCAE v5.0 toxicities. Even though only one study targeting extracting capecitabine toxicity is found, there are a lot of studies that utilize NLP methods to extract adverse drug events (ADEs) with other cancer drugs. For example, Fan et al. [33] developed a Bidirectional Encoder Representations from Transformers (BERT) based deep learning model to extract ADEs from open health forums (e.g., WebMD, Drugs.com). Although not specific to capecitabine, this study included a wide variety of anticancer drugs and demonstrated state-of-the-art performance (F1 = 0.97 for ADE extraction). Li et al. [34] developed an end-to-end deep learning model trained on the MADE 1.0 dataset [29], a publicly available corpus of expert-annotated electronic health records from oncology patients, specifically designed for extracting medication, indication, and ADE information. Their system combined a BiLSTM-CRF for NER and a BiLSTM-Attention mechanism for relation extraction, enabling high-quality extraction of medication-related events from clinical narratives. Our approach offers broader coverage than previous works, as we systematically compared the performance of rule-, machine learning, deep learning, and large language model (LLM) based methods within the same framework. Notably, to our knowledge, prior studies have not incorporated LLM-based methods for FP toxicity extraction, making our work the first to assess their potential in this context.

In this paper, our objective is to define, develop and evaluate multiple NLP systems, including rule-based, machine learning, deep learning and LLM-based systems, for capturing FP treatment and treatment-related toxicities from EHR clinical notes, and to identify the most effective approach. We have four contributions: (1) curate an annotated gold-standard corpus of 236 oncology notes; (2) perform a comparison of the performance of four NLP systems; (3) first to apply the error analysis prompting method to capture FP treatment and treatment-related toxicities and achieved the highest overall performance; (4) provide error analyses on 5 category and practical design to inform NLP pipelines for capturing FP treatment and treatment-related toxicities from unstructured EHR text.

## 2. Materials and methods

**Fig. 1.**
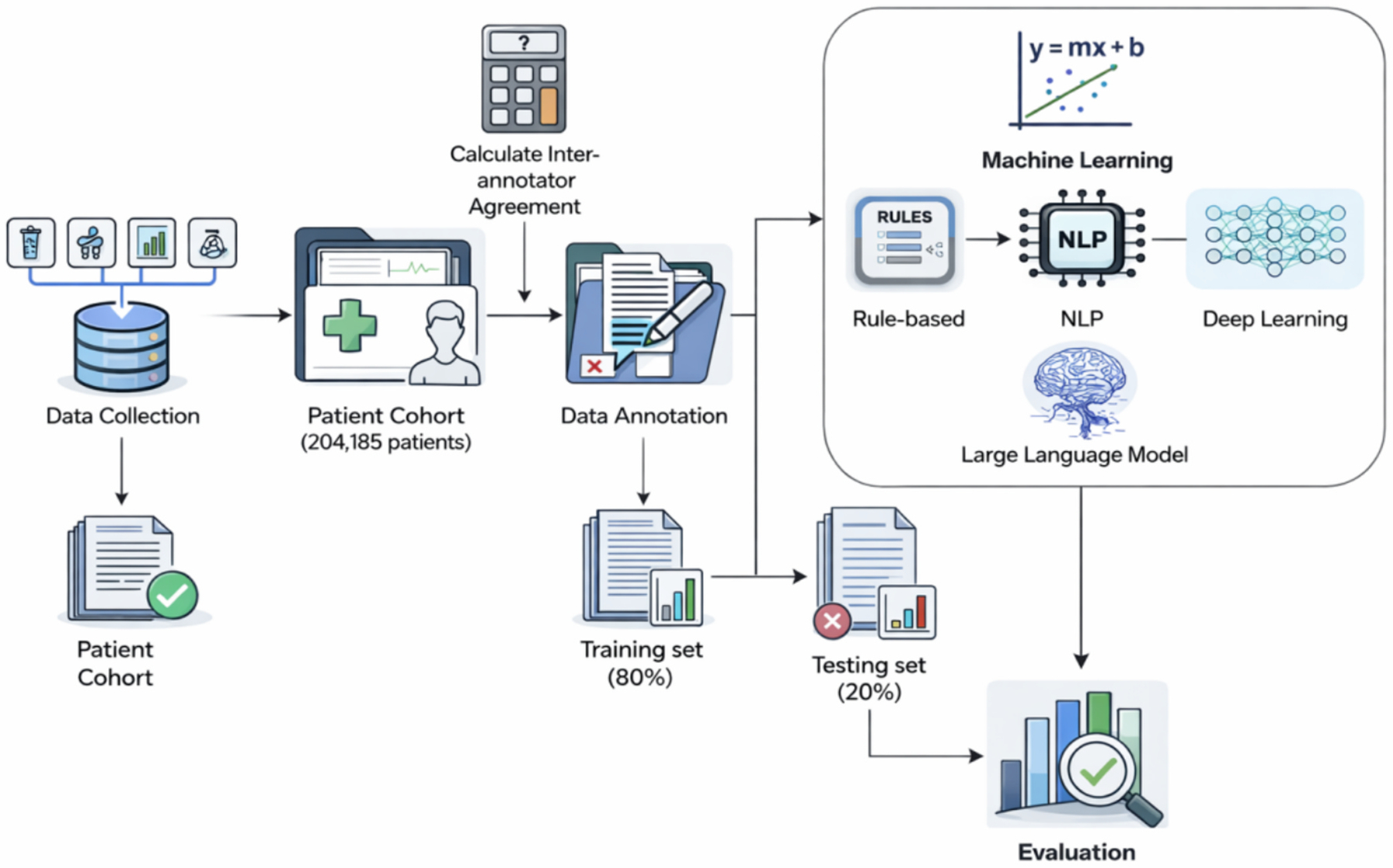
Workflow for the Extraction of Fluoropyrimidine Treatment and Treatment-related Toxicities.

Fig. 1 illustrates the workflow for the extraction of FP treatment and treatment-related toxicities. In the following subsections, we detail the data collection, FP treatment and treatment-related toxicity categories, and NLP approaches.

### 2.1. Data collection

We first identified a cohort of adult patients diagnosed with breast or gastrointestinal cancer between January 1, 2016 and December 31, 2022 at the University of Pittsburgh Medical Center (UPMC) through ICD-10 codes. Then patients with an EHR medication order for a fluoropyridine (i.e., capecitabine, 5-FU) from the UPMC health system between January 1, 2026 and December 31, 2023 were included for downstream analysis. All de-identified clinical notes during this time-frame were aggregated and disseminated through the University of Pittsburgh Clinical and Translational Science Institute Health Record Research Request (R3). These notes were exported from multiple types of clinical documents, including discharge summaries, outpatient progress notes, and physician reports. The clinical notes selected for inclusion in the corpus were aligned with each patient’s capecitabine initiation date, ensuring that all notes analyzed were temporally relevant to the course of capecitabine therapy and its potential treatment-related toxicities. This study was reviewed and designated as exempt by the University of Pittsburgh Institutional Review Board (IRB).

### 2.2. Fluoropyrimidine treatment and toxicity category and annotation

A multidisciplinary team of clinical experts including a hematologist/oncologist, a cardio-oncologist, an oncology pharmacist, and a pharmacist clinical researcher served as domain experts to develop 35 term categories covering FP treatment information, cardiotoxicity, and HFS. Initial term lists were assembled using both expert input and the CTCAE v.5. Categories were then systemically expanded through the National Cancer Institute’s (NCI) Enterprise Vocabulary Services (EVS) Explore tool and UMLS to incorporate synonyms and related terminology [35]. All the 35 term categories are included in the Supplementary Materials.

To create our gold standard dataset, we randomly sampled 300 clinical notes from the eligible patient cohort. During the annotation process, 64 notes were excluded as ineligible due to insufficient content, ambiguous documentation, or lack of relevant FP treatment information, resulting in 236 annotated notes for model development and evaluation. The domain experts independently annotated an initial set of 36 notes to establish inter-annotator agreement, achieving an average pairwise Cohen’s kappa of 0.77, indicating substantial agreement. Following agreement calibration, each expert annotated 50 additional notes, contributing to the final gold standard dataset of 236 notes. This annotation workflow is illustrated in Fig. 1. The 236 eligible notes were split 80:20 for training and testing for each category. This sample size meets established criteria for binary classification: (1) exceeds the minimum 10 events per variable for stable logistic regression, (2) provides > 80 % statistical power to detect meaningful performance differences (Δ F1 = 0.15) between methods, and (3) demonstrates convergent learning curves and stable cross-validation performance (SD < 0.05). The binary nature of our classification task and focused feature set allow for robust model development within this sample size.

Five annotation categories had sufficient representation and were defined to represent FP treatment and toxicities. FP treatments were annotated under the category drug of interest, which included capecitabine and 5-fluorouracil, their brand names (e.g., Xeloda, Adrucil) and abbreviations including either FP (e.g., 5-FU, FOLFOX, CAPEOX). FP treatment-related toxicities were defined as conditions observed in the context of FP use and likely attributed to the medication. These were annotated under the categories of arrhythmia, HF, valvular complications and HFS treatment/prevention therapies. Arrhythmia is defined as an abnormal rhythm of the heart [36], and its category captures mentions of cardiac rhythm disturbances. Heart failure (HF) is a cardiotoxicity side effect of FPs [37] and its category captures mentions of HF (e. g., heart failure, congestive heart failure) and related conditions (e.g., edema, leg swelling). Valvular complications refer to abnormalities or dysfunctions of the heart valves [38] and its category capture structural abnormalities (e.g., bicuspid aortic valve) and functional abnormalities (e.g., tricuspid regurgitation). HFS treatment/prevention therapies [3] refers to treatment and preventive medications used for HFS and its category captures mentions of prescription and over-the-counter topicals used to reduce HFS incidence or severity. Their definition and keywords are listed in Table 1.

### 2.3. Rule-based NLP algorithm

We developed a rule-based NLP algorithm for the toxicities extraction using MedTagger, a clinical NLP tool based on the Unstructured Information Management Architecture (UIMA) framework. The Med-Tagger software is publicly available at GitHub (https://github.com/OHNLP/MedTagger). Clinical experts provided a list of keywords and synonyms for each toxicity concept from medical terminologies and ontologies. We then used 80 % of the gold standard dataset as training data to develop regular expression rules for the NLP algorithm. Med-Tagger facilitated the execution of these regular expression-based rules, allowing the algorithm to annotate and extract information from unstructured clinical text data.

We applied an 80:20 train-test split across all toxicity categories, and this split was kept consistent across all methods. We specified customized negation rules based on patterns observed in our training data. These context rules handle various linguistic patterns including negation markers, uncertainty indicators, historical references, and experiencer identification. The regular expression patterns for toxicity concepts were developed to capture drug of interest, arrhythmia, HF, valvular complications, and HFS treatment/prevention therapies. The rule-based NLP algorithm including the customized negation rules can be found in the Supplemental Materials (Table S1).

### 2.4. Machine learning algorithms

We implemented a machine learning approach for binary classification where separate models were trained for each toxicity category. This design choice allows for independent optimization of each toxicity type and better handling of class imbalance issues that are common in medical datasets.

The machine learning approach contains three different machine learning algorithms for comparison: RF, SVM and logistic regression (LR). The approach follows a four-step workflow. First, data preprocessing is performed, which involves tokenizing sentences, converting text to lowercase, and removing non-informative symbols. Second, vectorization is used to transform text into numerical features for model input. SVM and LR use term frequency–inverse document frequency (TF-IDF) [39] features with sublinear term scaling. RF uses count-based n-gram features [40], which allow tree-based methods to exploit raw term frequencies without the normalization imposed by TF-IDF. Third, model training is conducted with class weighting set to balanced to address label imbalance. Fourth, validation is performed on test set and report precision, recall, F1 score for downstream comparison. We process five specific toxicity categories: drug of interest, arrhythmia, HF, valvular complications, and HFS treatment/prevention therapies. For each category, one binary classifier was trained, resulting in 15 total models (5 categories × 3 algorithms).

### 2.5. Deep learning algorithms

We implemented a deep learning pipeline based on two models: BERT [41] and ClinicalBERT [42]. The deep learning pipeline follows a four-step workflow. First, we tokenize the input text using BERT vector tokenizer. Second, contextual embeddings are generated by passing the tokenized sequences through either BERT model or ClinicalBERT, with the latter pre-trained on biomedical and clinical text to better capture domain-specific terminology. Third, model training is conducted independently for each toxicity category to enable task-specific adaptation. Fourth, evaluation is performed on a testing set, converting probabilistic outputs to binary predictions, and reporting precision, recall, and F1 score.

### 2.6. Large language model-based approach

We implemented two prompting strategies using LLaMA 3.1 8B: zero-shot prompting [39] and error analysis prompting [43]. We performed local inference using Ollama [46] to host LLaMA 3.1 8B, with temperature set to 0 and a context length of 8,192 tokens.

#### Zero-shot Prompting:

Zero-shot prompting performs binary classification without training examples. Each prompt contains three components: (1) the classification task description, (2) domain-specific medical terminology lists, and (3) explicit instructions for binary (“yes”/“no”) responses with explanations. For example, the HFS treatment/prevention prompt includes comprehensive lists of topical agents, while the arrhythmia prompt contains rhythm disturbance terminology. Table 2 shows the Zero-shot prompt used in this study.

#### Error Analysis Prompting:

**Fig. 2.**
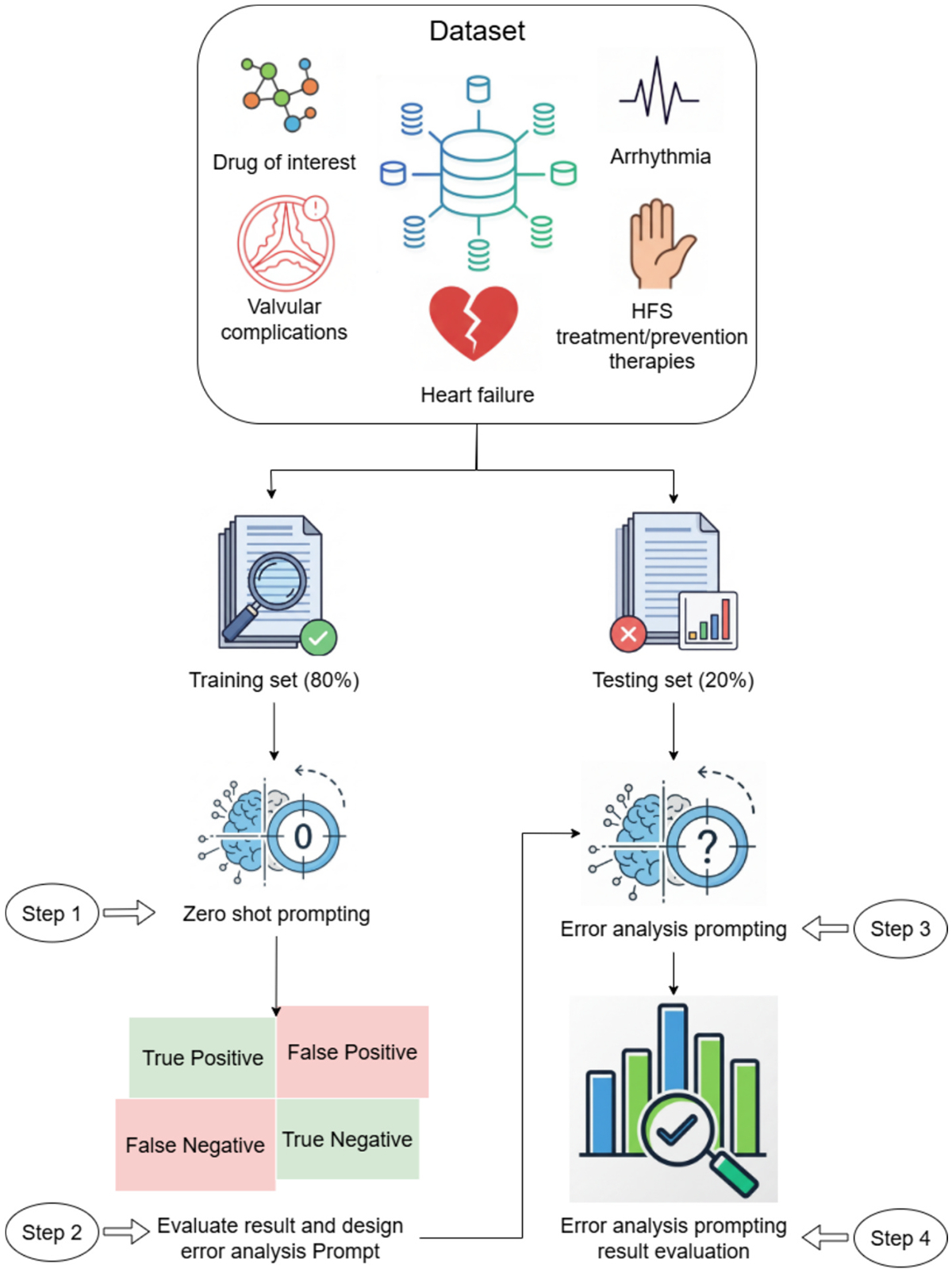
Error Analysis Prompting LLM.

Error analysis prompting enhances zero-shot performance by incorporating chain-of-thought (CoT) reasoning [44] derived from systematic error analysis of the results from the training dataset. Fig. 2 illustrates the four-step process. 1) Error Identification: We applied zero-shot prompting to the training set and systematically cataloged misclassifications, identifying recurring error patterns for each toxicity category. 2) Prompt Enhancement: False positive and false negative cases from Step 1 were analyzed to create corrective examples. The enhanced prompt combines: (a) original zero-shot instructions, and (b) CoT reasoning examples that demonstrate correct classification of previously misclassified cases. 3) Test Set Application: The error analysis enhanced prompts were applied to the held-out test set for final classification. 4) Performance Evaluation: Model outputs were evaluated against gold-standard annotations to calculate performance metrics.

Each CoT reasoning example follows a structured four-part analysis: (1) systematic parsing of clinical text, (2) identification of medical concepts, (3) matching against reference terminology, and (4) evidence-based classification decision. Table 2 provides complete examples of both prompting approaches and the CoT reasoning prompts are provided in the Supplemental Materials (Table S2).

To illustrate this process (as shown in Fig. 2), consider heart failure detection. The zero-shot prompt initially misclassified “bilateral lower extremity edema” as negative. Through error analysis (Step 1 in Fig. 2), we identified this pattern and created a CoT example (Step 2 in Fig. 2) demonstrating that bilateral edema represents fluid overload, a key heart failure indicator. This corrective reasoning was incorporated into the enhanced prompt, teaching the model to recognize indirect clinical manifestations.

The error analysis approach systematically addresses category-specific challenges. For arrhythmia, examples demonstrate distinguishing rhythm disturbances from general cardiac conditions. For valvular complications, examples clarify the difference between functional and structural abnormalities. This targeted error correction enables the model to handle complex clinical language patterns identified through training set analysis.

### 2.7. Evaluation

We use standard performance metrics, including precision, recall, and F1 score. Because the toxicity categories are imbalanced, we report weighted-averaged metrics as our primary summary. To obtain more reliable estimates given the limited dataset size, we evaluate all methods using the same 5-fold cross-validation with strictly disjoint training and test splits in each fold. For stochastic methods (machine learning, deep learning, and LLM-based approaches), we repeat each fold-level experiment 10 times (with different random seeds where applicable) and report the fold-level metric as the mean across the 10 runs. The rule-based method is deterministic and is not repeated with 10 runs. To quantify uncertainty, we report 95 % confidence intervals for precision, recall, F1, and accuracy using Student’s t-intervals computed over the 5-fold level scores (where each fold score is the mean over runs for stochastic methods). We use the t-distribution because the number of fold-level estimates is small and the variance is unknown. For the error-analysis prompting method, we first run the zero-shot prompt on the training set of the same fold and collect only training-set errors. We then summarize these errors into revised prompt instructions (i.e., an in-context prompt refinement) and apply the resulting prompt only to that fold’s held-out test set. We repeat this for each fold to avoid data leakage. This fold-specific evaluation ensures that the test data remain fully unseen and prevents leakage.


$$\text{Precision}=\frac{\text{True}\;\text{Positive}}{\text{True}\;\text{Positive}+\text{False}\;\text{Positive}}$$

$$\text{Recall}=\frac{\text{True}\;\text{Positive}}{\text{True}\;\text{Positive}+\text{False}\;\text{Negative}}$$

$$F1\;\text{score}=\frac{2\times \text{Precision}\times \text{Recall}}{\text{Precision}+\text{Recall}}$$

## 3. Results

The performance of the rule-based NLP algorithm, machine learning, and LLMs in extracting FP treatment and treatment-related toxicity is presented in Table 3. The error analysis prompting approach using LLaMA 3.1 8B achieved the optimal overall performance with a F1 score of 1.000 in FP treatment and a F1 score of 0.965 treatment-related toxicity. This result demonstrates the effective generalization capabilities of LLMs when provided with task-specific examples. The model was able to match expert-level annotation, suggesting promising potential for practical clinical NLP applications. The zero-shot prompting method using LLaMA 3.1 8B also showed robust performance, particularly in the Arrhythmia toxicity extraction, where it reached F1 score of 0.970. While it did not match the error analysis prompting optimal scores, its performance was still high in FP treatment (F1 = 0.889), Valvular Complications (F1 = 0.829) and HFS treatment/prevention therapies (F1 = 0.891), further validating the utility of LLMs prompting even without fine-tuning or in-context examples. However, the zero-shot prompting approach did not do well for HF (F1 = 0.727).

For deep learning-based NLP algorithms leveraging pre-trained language models, ClinicalBERT outperformed the base BERT model in extracting some treatment-related toxicities, such as Arrhythmia (F1 = 0.901), Heart Failure (F1 = 0.854) and Valvular Complications (F1 = 0.822). Both BERT-based models reached consistent F1 scores (ClinicalBERT F1 = 0.797 and BERT F1 = 0.792) for FP Treatment extraction and performed similarly in the HFS treatment/prevention therapies category (ClinicalBERT F1 = 0.960 and BERT F1 = 0.967). However, BERT-based models struggled with Heart Failure, where F1 scores of 0.768 and confidence interval in [0.674, 0.862]. BERT model yields the lowest average performance in treatment-related toxicities extraction (F1 = 0.837). This suggests that these categories may require additional domain-specific adaptation for BERT-based models.

Among traditional approaches, the rule-based NLP system served as our baseline, achieving a F1 score of 1.000 in FP Treatment extraction and an average F1 score of 0.904 in treatment-related toxicities extraction. It performed particularly well on Arrhythmia (F1 = 0.938) and Heart Failure (F1 = 0.967), outperforming SVM, RF and deep learning methods on these categories. This suggests that when domain knowledge is available, rule-based systems can remain competitive with machine learning and deep learning methods. Machine learning models like LR and SVM also delivered robust results in treatment-related toxicities extraction (LR F1 = 0.914, SVM F1 = 0.903). It is noteworthy that LR and SVM yielded similar results, which may be attributed to the small validation set and the similarity of the two algorithms. Both SVM (with a linear kernel) and logistic regression are linear classifiers that aim to identify optimal separating hyperplanes. On small, linearly separable datasets, they frequently converge to similar solutions. LR and SVM models performed best in HFS treatment/prevention therapies (LR F1 = 0.976, SVM F1 = 0.984). However, performance on HF was noticeably lower (LR F1 = 0.809, SVM F1 = 0.760), which may be attributed to class imbalance and variability in language in this category.

Lastly, the RF classifier yielded the lowest performance in extracting FP treatment with a F1 score of 0.745. Among treatment-related toxicities, it struggled particularly in identifying HF (F1 = 0.744), indicating that tree-based models may lack the flexibility to capture complex patterns in clinical narratives without substantial feature engineering.

## 4. Discussion

In this study, we compared rule-based, machine learning, deep learning, and LLM–based methods for extracting FP treatment and treatment-related toxicities from oncology clinical notes. Error analysis prompting approach achieved the highest overall performance, indicating that when guided by carefully constructed prompts incorporating representative error cases, LLMs can match expert-level annotation even in specialized clinical domains. Zero-shot prompting performed moderately, underscoring the potential for rapid deployment without fine-tuning. Rule-based methods performed competitively, particularly for the capecitabine, arrhythmia, heart failure and valvular complications category, reflecting their continued value in settings with limited training data. Traditional machine learning and deep learning models were effective for most categories in treatment-related toxicity, but they struggled with the Heart Failure category. This is because many heart failure findings are indirect (e.g. leg edema), highlighting the challenge of extracting conditions expressed through indirect clinical findings.

The toxicity categories in our study show strong conceptual alignment with those in Hong et al. [30], as both were anchored to CTCAE v5.0 terminology (e.g., arrhythmia, heart failure). This overlap supports the comparability of our results and enhances the validity of our findings.

We observed occasional perfect F1 scores in our results. Even though EHR are often noisy, our gold-standard labels are sentence-level clinician annotations rather than document-level inferences. The annotation guidelines for each category are primarily driven by the presence of category-specific keywords or phrases within one sentence, with limited reliance on cross-sentence context or multi-step clinical reasoning. As a result, our task achieved a few perfect F1 scores (F1 = 1.000) when these cues are strong and consistently annotated.

This study has several limitations. First, the dataset is small (236 annotated notes) and drawn from a single institution. Even though a 5 fold cross validation is used to improve the robustness of our models the results may not generalize to other healthcare settings or EHR systems. Second, we focused on a single drug class (FPs) and a subset of toxicity categories, which may not fully represent the complexity of oncology drug toxicities. Third, our final terminology set was constrained by the initial development corpus, which limited the discovery of additional clinically relevant toxicity terms and may restrict model prediction and generalizability. Fourth, our extraction task is sentence-level and therefore does not evaluate document-level contextual linking, such as relationships between treatment exposure and toxicity mentions; this will be addressed in the future work.

## 5. Conclusion

In this study, we developed and compared rule-based, machine learning, deep learning, and LLM-based NLP systems for FP treatment and treatment-related toxicities extraction from oncology clinical notes. Our findings demonstrate that error analysis prompting with LLM achieved the highest overall performance. Error analysis prompting and zero-shot prompting achieved the best performance in FP Treatment extraction. Machine Learning methods achieved second best performance in treatment-related toxicities extraction. Deep learning based methods underperformed and were most challenged by heart failure categories.

These findings suggest that carefully designed prompting can enable LLM to match expert-level annotation. Our results demonstrate the promise of LLM-based prompting-enhanced by error-analyzed examples-for extraction of FP treatment and treatment-related toxicities from oncology clinical notes.

## 6. Future work

Future research should prioritize validating these methods in independent cohorts of patients receiving fluoropyrimidines for gastrointestinal and breast cancers. Multi-institutional validation across diverse healthcare systems would assess generalizability of model performance and robustness to variations in clinical documentation practices. Testing in different geographic regions and EHR platforms would further establish the reliability of our approach for fluoropyrimidine toxicity extraction.

Methodological improvements should focus on automated prompt optimization for LLMs and exploration of additional architectures to enhance interpretability and reliability. Integration of structured data elements could improve toxicity detection, though the utility varies by toxicity type. While cardiotoxicity manifestations have corresponding ICD-10 codes (e.g., I48 for atrial fibrillation, I50 for heart failure), medication-induced hand-foot syndrome lacks specific diagnostic codes, highlighting the particular importance of NLP extraction for toxicities without structured coding options. Combining NLP with available structured data—including laboratory values, vital signs, and relevant ICD codes where applicable—could enhance overall system performance.

Temporal modeling to capture the relationship between drug exposure and toxicity onset represents another important direction, as our current approach does not explicitly model time-dependent relationships. Development of a standardized fluoropyrimidine toxicity ontology would improve consistency across institutions and facilitate integration into clinical decision support systems [45]. Such an ontology could harmonize toxicity definitions, severity grading, and documentation standards, enabling more reliable cross-institutional research and real-world evidence generation.

Finally, evaluation in clinical research settings would assess the impact of this approach on advancing the understanding of treatment-related toxicities. Reliable and scalable extraction could improve characterization of adverse events, guide future studies on toxicity mechanisms, and inform strategies for treatment optimization and prevention in patients receiving FPs.

## Supplementary Material

Supplemental Materials

## Figures and Tables

**Table 1:** FP treatment and treatment-related toxicity.

	Category	Definition	Keywords
Fluoropyrimidine Treatment	Drug of interest	Mentions of capecitabine or 5-FU, including brand names, combination therapies and abbreviations	Capecitabine, Xeloda, Xitabin 5-FU, 5-Fluorouracil, Fluoro Uracil, Adrucil, Carac, Flurablastin CAPOX, CAPIRI, CAPEOX, CAPEMONO, FOLFOX, FOLFIRI, FOLFOXIRI, MFOLFOX, AIO, De Gramont Regimen, XELOX, XELIRI, FOLFIRINOX.
Treatment-related Toxicity	Arrhythmia	An abnormal rhythm of the heart	Cardiac Arrhythmia, Dysrhythmia, Irregular Heartbeat, Heart Rhythm Disturbance, Cardiac Rhythm Disorder Afib, A Fib, Atrial Flutter, Auricular Flutter, A-flutter, AF, Auricular Fibrillation VF, Ventricular Fibrillation, Cardiac Arrest Due to VF, Ventricular Tachycardia (V TACH), Tachycardia; Ventricular Flutter Atrial, Fibrillation Atrial, Flutter Auricular, Heart Arrhythmia
	Heart failure	Mentions of heart failure and related conditions associated with capecitabine use.	HF, Cardiac Failure, Heart Insufficiency, Myocardial Failure, Cardiac Insufficiency, Bilateral Leg Edema, Swelling, Dropsy, Hydrops, Oedema, Fluid Overload Reduced Ejection Fraction (EF or LVEF), Reduced LV Function Cardiogenic Shock, Heart Shock, Cardiovascular Collapse, HF Exacerbation.
	Valvular complications	The structural abnormalities and functional abnormalities of the heart valves associated with capecitabine use.	TR, Tricuspid Insufficiency, Incompetence, Right AV Valve Regurgitation AR, Aortic Regurgitation, Aortic Incompetence, Aortic Valve Insufficiency Valve Disorder, AV Valve Abnormality, Valvular Dysfunction.
	HFS treatment/prevention therapies	The mentions of topical measures used to reduce HFS severity or incidence	Cream, Urea Cream, Aqua Care, Nutraplus, Vanamide, Carbamide, Elaqua XX, Lanaphilic, Ureaphil, Carbamide, Utterly Smooth, Udderly Smooth Cream, Lotion, Gel, Ointment, Salve, Solution, Suspension Uridine Triacetate (Vistogard)

**Table 2:** Example of zero-shot prompt and error analysis prompt on heart failure.

Zero-shot Prompt	You are given a sentence from a clinical text, if that sentence contains any information related to instances of HF, respond with yes and explain why. If not, respond with no and explain why. These words being the signs and evidence: HF, cardiac failure, heart insufficiency, myocardial failure, cardiac insufficiency bilateral leg edema, swelling, dropsy, hydrops, oedema, fluid overload reduced ejection fraction (EF or LVEF), reduced LV function cardiogenic shock, heart shock, cardiovascular collapse, HF exacerbation. If these words are mentioned in the sentence, respond with yes and explain why. If these words are not mentioned in the sentence, respond with no and explain why.
Error Analysis Prompt	You are given a sentence from a clinical text, if that sentence contains any information related to instances of HF, respond with yes and explain why. If not, respond with no and explain why. These words being the signs and evidence: HF, cardiac failure, heart insufficiency, myocardial failure, cardiac insufficiency bilateral leg edema, swelling, dropsy, hydrops, oedema, fluid overload reduced ejection fraction (EF or LVEF), reduced LV function cardiogenic shock, heart shock, cardiovascular collapse, HF exacerbation. If these words are mentioned in the sentence, respond with yes and explain why. If these words are not mentioned in the sentence, respond with no and explain why. Here is a sentence: Trace edema bilateral lower extremities. Reasoning: Step 1: Read the sentence and identify key terms that may indicate signs or evidence of HF.” Step 2: The phrase “bilateral lower extremities edema” directly matches one of the listed indicators: “bilateral leg edema”, which is a known clinical sign of fluid overload.Step 3: Although the sentence does not explicitly mention a diagnosis of HF, bilateral edema is a common and recognized symptom associated with congestive heart failure (CHF). Step 4: Since the sentence includes a relevant physical finding from the keyword list, it provides indirect evidence consistent with possible HF. Answer: Yes. The sentence mentions “bilateral lower extremities edema,” which matches “bilateral leg edema,” a recognized sign suggestive of HF or fluid overload.

**Table 3:** 5-Fold Cross-validation performance of rule-based, machine learning, deep learning, and LLM-based methods for FP treatment and treatment-related toxicity extraction (10 runs per fold and averaged).

	FP Treatment	Treatment-related Toxicity				
Precision	Capecitabine	Arrhythmia	Heart Failure	Valvular Complications	HFS treatment/ prevention therapies	Average
Recall	(n = 8)	(n = 14)	(n = 11)	(n = 9)	(n = 25)	
F1 score						
Rule-based NLP	**1.000 [1.000, 1.000]**	0.975 [0.906, 1.000]	**0.943 [0.784, 1.000]**	1.000 [1.000, 1.000]	1.000 [1.000, 1.000]	0.979 [0.947, 1.000]
	**1.000 [1.000, 1.000]**	0.910 [0.806, 1.000]	**1.000 [1.000, 1.000]**	0.880 [0.658, 1.000]	0.646 [0.541, 0.752]	0.859 [0.781, 0.937]
	**1.000 [1.000, 1.000]**	0.938 [0.893, 0.982]	**0.967 [0.874, 1.000]**	0.928 [0.791, 1.000]	0.782 [0.704, 0.861]	0.904 [0.856, 0.951]
SVM	0.868 [0.738, 0.998]	0.940 [0.868, 1.000]	0.820 [0.718, 0.922]	0.967 [0.874, 1.000]	0.985 [0.960, 1.000]	0.928 [0.887, 0.969]
	0.839 [0.699, 0.980]	0.925 [0.833, 1.000]	0.776 [0.642, 0.910]	0.950 [0.811, 1.000]	0.984 [0.957, 1.000]	0.909 [0.856, 0.962]
	0.836 [0.691, 0.980]	0.923 [0.828, 1.000]	0.760 [0.593, 0.926]	0.947 [0.799, 1.000]	0.984 [0.957, 1.000]	0.903 [0.844, 0.962]
LR	0.831 [0.647, 1.000]	0.935 [0.850, 1.000]	0.836 [0.736, 0.936]	0.967 [0.874, 1.000]	0.978 [0.952, 1.000]	0.929 [0.890, 0.968]
	0.786 [0.590, 0.982]	0.926 [0.834, 1.000]	0.813 [0.720, 0.905]	0.950 [0.811, 1.000]	0.976 [0.948, 1.000]	0.916 [0.871, 0.961]
	0.779 [0.581, 0.978]	0.926 [0.833, 1.000]	0.809 [0.716, 0.902]	0.947 [0.799, 1.000]	0.976 [0.948, 1.000]	0.914 [0.868, 0.960]
RF	0.843 [0.708, 0.979]	0.917 [0.857, 0.977]	0.854 [0.749, 0.960]	0.916 [0.812, 1.000]	0.933 [0.884, 0.983]	0.905 [0.873, 0.937]
	0.775 [0.584, 0.965]	0.893 [0.816, 0.970]	0.771 [0.594, 0.948]	0.871 [0.705, 1.000]	0.918 [0.850, 0.986]	0.863 [0.810, 0.916]
	0.745 [0.521, 0.969]	0.891 [0.811, 0.970]	0.744 [0.540, 0.948]	0.862 [0.683, 1.000]	0.917 [0.847, 0.986]	0.853 [0.794, 0.913]
BERT	0.835 [0.690, 0.980]	0.885 [0.825, 0.945]	0.814 [0.705, 0.923]	0.813 [0.672, 0.954]	0.971 [0.952, 0.990]	0.871 [0.826, 0.916]
	0.804 [0.644, 0.964]	0.854 [0.776, 0.931]	0.784 [0.699, 0.869]	0.775 [0.640, 0.909]	0.967 [0.944, 0.989]	0.845 [0.797, 0.893]
	0.792 [0.622, 0.963]	0.849 [0.768, 0.930]	0.768 [0.674, 0.862]	0.764 [0.625, 0.904]	0.967 [0.944, 0.989]	0.837 [0.786, 0.888]
ClinicalBERT	0.844 [0.677, 1.000]	0.921 [0.865, 0.977]	0.872 [0.759, 0.985]	0.854 [0.752, 0.956]	0.964 [0.942, 0.987]	0.903 [0.868, 0.938]
	0.805 [0.604, 1.000]	0.903 [0.833, 0.973]	0.857 [0.739, 0.975]	0.826 [0.694, 0.957]	0.961 [0.936, 0.985]	0.886 [0.845, 0.928]
	0.797 [0.588, 1.000]	0.901 [0.827, 0.974]	0.854 [0.733, 0.975]	0.822 [0.684, 0.960]	0.960 [0.936, 0.985]	0.884 [0.842, 0.927]
Zero-Shot Prompting	0.906 [0.872, 0.939]	0.970 [0.931, 1.000]	0.817 [0.738, 0.897]	0.877 [0.820, 0.935]	0.905 [0.861, 0.950]	0.893 [0.860, 0.926]
	0.892 [0.860, 0.925]	0.970 [0.931, 1.000]	0.750 [0.630, 0.870]	0.837 [0.762, 0.912]	0.893 [0.839, 0.947]	0.863 [0.815, 0.910]
	0.889 [0.850, 0.928]	0.970 [0.931, 1.000]	0.727 [0.573, 0.882]	0.829 [0.734, 0.924]	0.891 [0.835, 0.947]	0.854 [0.800, 0.908]
Error Analysis Prompting	**1.000 [1.000, 1.000]**	**0.975 [0.906, 1.000]**	1.000 [1.000, 1.000]	**1.000 [1.000, 1.000]**	**1.000 [1.000, 1.000]**	**0.994 [0.981, 1.000]**
	**1.000 [1.000, 1.000]**	**1.000 [1.000, 1.000]**	0.800 [0.800, 0.800]	**1.000 [1.000, 1.000]**	**0.973 [0.926, 1.000]**	**0.943 [0.902, 0.984]**
	**1.000 [1.000, 1.000]**	**0.987 [0.950, 1.000]**	0.889 [0.889, 0.889]	**1.000 [1.000, 1.000]**	**0.986 [0.961, 1.000]**	**0.965 [0.943, 0.988]**

## Data Availability

The data used in this study are electronic health records that may contain sensitive protected health information (PHI). Thus, it is not available for public access. The NLP algorithms are publicly available at GitHub: https://github.com/PittNAIL/NLP4FPandToxicity.

## Appendix A. Supplementary data

Supplementary data to this article can be found online at https://doi.org/10.1016/j.ijmedinf.2026.106276.
