# Supplementary material for: Automated extraction of fluoropyrimidine treatment and treatment-related toxicities from clinical notes using natural language processing
Source: Int J Med Inform. Author manuscript; Available in PMC 2026 Apr 1. (PMC13041774; doi:10.1016/j.ijmedinf.2026.106276)
Supplement: Supplemental Materials [file NIHMS2160658-supplement-Supplemental_Materials.docx]

**Supplementary materials**

**Table S1. The rule-based NLP algorithm for the extraction of fluoropyrimidine treatment and treatment-related toxicities**

| Category | Keywords |
| --- | --- |
| Drug of interest | Capecitabine, Xeloda, Xitabin 5-FU, 5-Fluorouracil, Fluoro Uracil, Adrucil, Carac, Flurablastin CAPOX, CAPIRI, CAPEOX, CAPEMONO, FOLFOX, FOLFIRI, FOLFOXIRI, MFOLFOX, AIO, De Gramont Regimen, XELOX, XELIRI, FOLFIRINOX. |
| HFS treatment/prevention therapies | Cream, Urea Cream, Aqua Care, Nutraplus, Vanamide, Carbamide, Elaqua XX, Lanaphilic, Ureaphil, Carbamide, Utterly Smooth, Udderly Smooth Cream, Lotion, Gel, Ointment, Salve, Solution, Suspension Uridine Triacetate (Vistogard) |
| Treatment modifiers | Capecitabine induced, Fluoropyrimidine induced, FP induced, Xeloda induced, 5-FU induced, Chemo induced, Chemotherapy induced |
| Treatment doses | 14 ON, 7 ON, 14 OFF, 7 OFF, 1-14 DAYS ON, 1-14 ON, 1-14 DAYS OFF, 1-14 OFF, BID, twice daily, once daily, 1-2 time(s) a day |
| Radiation | Chemoradiation, Chest radiation |
| Treatment Outcomes | Dose decrease, decrease dose, discontinue, Discontinuation, delay, hold, dose reduction, reduce dose, reduce frequency, frequency reduction, stop, start, initiate, initiation, begin, began, started, day 1, cycle 1 |
| HFS | Acral Erythema, Chemotherapy-Induced, Acral Erythemas, Chemotherapy-Induced, Chemotherapy Induced Acral Erythema, Chemotherapy Induced Palmoplantar Erythrodysesthesia, Chemotherapy-Induced Acral Erythema, Chemotherapy-Induced Acral Erythemas, Chemotherapy-Induced Palmoplantar Erythrodysesthesia, Chemotherapy-Induced Palmoplantar Erythrodysesthesias, Hand and foot syndrome secondary to chemotherapy, Hand Foot Syndrome, Hand-Foot Syndrome, Hand-Foot Syndromes, Palmar-plantar erythrodysaesth., Palmar-plantar erythrodysaesthesia syndrome, palmar-plantar erythrodysesthesia, Palmar-plantar erythrodysesthesia syndrome, Palmar-Plantar Erythrodysthesia, Palmoplantar Erythrodysesthesia, Chemotherapy-Induced, Palmoplantar Erythrodysesthesias, Chemotherapy-Induced, Syndrome, Hand-Foot, Syndromes, Hand-Foot, palmar-plantar erythrodysesthesia syndrome (diagnosis), hand-foot, HFS, CiHFS, palmar plantar erythrodysthesia, PPE, palmar-plantar, hand and foot, hand and foot syndrome, L27.1, capecitabine-induced hand-foot syndrome |
| Skin Integrity | Peeling, Peeling of skin, Desquamation, Desquamative state, Scaling, Dropping of scales, Exfoliating, Flaking of skin, Shedding of scales, Exfoliation, Scaly skin, peeling skin, flaking skin, Skin exfoliation, Skin peeling, Skin desquamation, Exfoliation of skin, Scaling of skin, Skin scaling, Scaling of the skin, skin appearance peeling, skin scales, scales, peeling skins, skin peel, exfoliate, peel, skin flaking, desquamation; skin, Peeling;skin, Desquamation of skin, Blisters, skin vesicle, Have Blistering, Has blistering, Blister of skin, Cutaneous blister, Dermal blister, Skin blister, Skin blisters, Skin blistering, Blistering skin, blister skin, Bleeding skin, bleeding of skin, Skin bleeding, bleed skin, bleeds skin, Fissures, Skin Fissure, Skin fissuring (palms), Skin fissuring (soles), Fissure in skin, Cracks in skin, Splits in skin, Cracked skin, Fissuring of skin, cracking of skin, skin crack |
| Edema | Edema of palm of hand, edema of left palm of hand, edema of bilateral palm of hand, edema of right palm of hand, Edema of sole of foot |
| Hyperkeratosis | Grade 1 Hyperkeratosis, Increased Keratinization, Grade 3 Hyperkeratosis, Excessive cornification, Keratosis, Tylosis, Hyperkeratoses, hyperkeratosis of skin, skin texture, hyperkeratosis, skin hyperkeratosis |
| Pain | Pain of left hand, pain in left hand only, Pain in right hand, pain in right hand only, limb pain right hand, limb pain left hand, right hand pain, Hand pain, Painful hand, Pain in hand, Pain in limb, hand, pain in the hands, limb pain hand, hands pain, Pain;hand, soft tissue pain in foot, foot pain, Burning pain in hand, Burning pain in foot, Skin symptom change, Skin change |
| Dermatitis | Dermatitides, Skin inflamed, Skin inflammation, Inflammation of Skin, skin; inflammation, inflammation; skin, Skin—Inflammation |
| Erythema/Redness | Erythema of skin, Cutaneous redness, Dermal erythema, Skin reddened, Skin red, Skin erythema, erythematous condition, unusual change in color of skin to red (erythema), redness of skin, skin redness, erythema findings, Erythematous conditions, Erythematous disorder, Red skin |
| Cardiotoxicity | Cardiac Toxicity, Cardiotoxicities, Toxicity; Cardiac, Cardiac Toxicities |
| Myocardial Infarction | Heart Attack, Infarction; Myocardial, Myocardial Infarcts, Heart Attacks, Cardiac infarction, MI, Infarction of heart, Attack coronary, Right ventricular infarction, Infarct myocardial, myocardial necrosis, infarctions myocardial, heart infarction, attack heart, attacking heart, attacks coronary, coronary attack, disorder infarction myocardial, syndrome myocardial infarction, infarction; myocardial, cardiac; infarction |
| Cardiovascular Strokes | Stroke; Cardiovascular, Strokes; Cardiovascular, Cardiovascular Stroke |
| Heart failure | Cardiac Failure, HF, Cardiac insufficiency, Weak heart, Cardiac function fail, Heart insufficiency, Failure heart, failures heart, myocardial failure, heart weak, hearts weak, insufficiency heart, failure; heart, cardiac; failure, cardiac; insufficiency, failure; cardiac, heart; insufficiency, insufficiency; cardiac, insufficiency; heart, weak; heart, Failure;heart, Failure;cardiac, Insufficiency;cardiac, Weakness;heart, HEART FAILURE AND OTHER FUNCTIONAL DISORDERS, BLE edema, Cardiac insufficiency, Interstitial edema, Edematous, Oedema, Waterlogged, Dropsy, Hydrops, swelling due to excess fluid, excess fluid, Bilateral lower extremity edema, Exacerbation of congestive heart failure, congestive heart failure exacerbation, CHF exacerbation, Reduced left ventricular ejection fraction, Reduced ejection fraction, Reduced EF, Reduced LVEF, Reduction in EF, Cardiogenic Shock, Shock, Cardiogenic, Cardiovascular shock, Shock cardiogenic, heart shocking, heart shock, shock heart, cardiogenic; shock, shock; cardiogenic, Cardiac shock syndrome, Power failure syndrome, cardiocirculatory collapse, HF exacerbation |
| Angina | Angina Pectoris, Angor Pectoris, Stenocardia, Ischemic chest pain, Anginal syndrome, Cardiac angina, AP - Angina pectoris, Ischaemic chest pain, Anginal pain, Anginal discomfort, chest pain, Ischemic heart disease with angina, chest; pain, ischemic, pain; chest, ischemic, syndrome; anginal, anginal; syndrome, Pain;angina, ANGINAL SYNDROMES, Angina of effort, Pain in chest, Chest pain, Pain, Chest, Pains, Chest, Thorax painful, Thoracic pain, Thorax pain, Thoracalgia, Stethalgia, chest pain or discomfort, Chest discomfort, Discomfort in chest, pain thoracic, chest; pain, pain; chest, pain; thorax, thorax; pain, Pain;chest, Heart throbbing, heart irregularities, heart throb, Cardiac angina syndrome |
| Arrhythmia | Cardiac Arrhythmia, Dysrhythmia, Irregular Heartbeat, Heart Rhythm Disturbance, Cardiac Rhythm Disorder Afib, A Fib, Atrial Flutter, Auricular Flutter, A-flutter, AF, Auricular Fibrillation VF, Ventricular Fibrillation, Cardiac Arrest Due to VF, Ventricular Tachycardia (V TACH), Tachycardia;Ventricular Flutter Atrial, Fibrillation Atrial, Flutter Auricular, Heart Arrhythmia |
| Coronary Artery Vasospasms | Coronary Vasospasm, Coronary Artery Spasm, Artery Vasospasm, Coronary, Vasospasm, Coronary Artery, Coronary Vasospasms, Vasospasm, Coronary, Artery Spasm, Coronary, Coronary Artery Spasms, Spasm, Coronary Artery, Coronary spasm, Arteriospasm coronary, Coronary vascular spasm, Spasm coronary artery, artery coronary spasms, coronaries spasm, vasospasm coronary, a.coronaria; spasm, coronary; spasm, spasm; coronary, Spasm;artery;coronary |
| Dyspnea | Shortness of Breath, Breath Shortness, Difficulty breathing, Respiration difficult, DIB - Difficulty in breathing, SOB - Shortness of breath, Breathless, Dyspnoea, Abnormal breathing, Difficult to breathe, Trouble breathing, Breathing difficult, SOB (shortness of breath), tightness of breath, s.o.b. |
| Syncope | Fainting, Syncope and collapse, Blackout, Passed out, Syncopal attack, swoon, loss of consciousness; attack, Syncopal episode |
| Cardiomyopathies | Myocardial Disease, Myocardiopathies, Cardiomyopathy, Myocardiopathy, Diseases, Myocardial, Disease, Myocardial, Disorder of heart muscle, Disorder of myocardium, Myocardiodystrophy, heart muscle disease, myocardium; disease, Myocardium--Diseases |
| Myocardial Ischemia | Ischemic Heart Disease, Ischemia, Myocardial, Heart Disease, Ischemic, Disease, Ischemic Heart, IHD - Ischemic heart disease, Cardiac ischemia, Ischemia myocardial, ischemia; heart, myocardium; ischemic, ischemia; myocardial, Disease;ischaemic heart, HEART, ISCHEMIC DISEASE, Myocarditides, Myocarditis, Myocardial inflammation, Inflammation of heart muscle, myocardium; inflammation, inflammation; myocardium |
| Pericarditis | Swelling or irritation of membrane around heart, an inflammation of the membrane surrounding the heart, pericardium; inflammation, inflammation; pericardium |
| Endocarditis | Endocarditides, inflammation of the heart valve |
| Cardiac Arrest | Heart Arrest, Asystole, Arrest, Cardiac, Arrest, Heart, Asystoles, Cardiac standstill, Heart stops beating, Ventricular asystole, Asystolia, Ventricular asystolia, Asystolic, Ventricular arrest, Arrest cardiac, Standstill cardiac, Cardiac arrest, unspecified, cardiac asystole, stoppage; heart, arrest; cardiac, heart; arrest, heart; stoppage, ventricular; arrest, arrest; ventricular, Cardiac arrest- asystole, CA - Cardiac arrest, Cardiopulmonary Arrest, Arrest, Cardiopulmonary, Cardiorespiratory arrest, Cardio-respiratory arrest, cardiac arrest cardiorespiratory, arrest cardiopulmonary, arrest cardio respiratory, cardiorespiratory; arrest, arrest; cardiorespiratory |
| Ventricular Dysfunction | Dysfunction, Ventricular, dysfunction ventricular |
| Pericardial effusion | Effusion, Pericardial, Fluid in pericardium, Fluid around heart, Effusion pericardial, Pericardial fluid, pericardium; effusion, effusion; pericardial, effusion; pericardium |
| Heart Block | Block, Heart, Block heart, Conduction block, blocked heart, blocks heart, blockage heart, blockages heart, cardiac; block, myocardial; block, block; heart, heart; block, block; cardiac, block; conduction, block; myocardial, conduction; block, Block;heart |
| Hypotension | Blood Pressure, Low, Low Blood Pressure, Hypopiesis, Blood pressure decreased, Arterial blood pressure decreased, Blood pressure drop arterial, Pressure arterial decreased, Drop of blood pressure, Fall in blood pressure, BP fell, Low BP, Blood pressure dropped, Drop in blood pressure, Blood pressure low, Lowered blood pressure, BP lowered, blood decreasing pressure, blood dropping pressure, blood drops pressure, blood falling pressure, blood falls pressure, blood lowered pressure, bp lower, decreased blood pressure, Hypotensive |
| Cardiac Tamponade | Pericardial Tamponade, Tamponade, Cardiac, Tamponade, Pericardial, Tamponade cardiac, Tamponade, tamponade; cardiac, HEART, TAMPONADE, Rose's tamponade, heart tamponade |
| Heart valve regurgitation | Valvular insufficiency, Cardiac valve insufficiency, Mitral Valve Insufficiency, MITRAL REGURGITATION, Mitral Insufficiency, Mitral Incompetence, Mitral Valve Incompetence, Valve Insufficiency, Mitral, Insufficiency, Mitral Valve, Regurgitation, Mitral, Regurgitation, Mitral Valve, Insufficiency, Mitral, MI - Mitral incompetence, MR - Mitral regurgitation, Regurgitation of left atrioventricular valve, Ventriculo-atrial regurgitation |
| Valvular complications | Tricuspid Valve Insufficiency, Tricuspid Valve Regurgitation, Tricuspid Incompetence, Tricuspid Regurgitation, Insufficiency, Tricuspid Valve, Valve Insufficiency, Tricuspid, Regurgitation, Tricuspid, Regurgitation, Tricuspid Valve, Valve Incompetence, Tricuspid, Incompetence, Tricuspid, Incompetence, Tricuspid Valve, TR - Tricuspid regurgitation, TI - Tricuspid incompetence, Regurgitation of right atrioventricular valve, heart valve disorder atrioventricular right leaflet, abnormality regurgitation, Aortic Valve Insufficiency, Aortic Valve Incompetence, Regurgitation, Aortic Valve, Aortic Regurgitation, Insufficiency, Aortic Valve, Incompetence, Aortic, Incompetence, Aortic Valve, Regurgitation, Aortic, Aortic valve regurgitation, Aortic insufficiency, AI - Aortic incompetence, AR - Aortic regurgitation, Aortic incompetence, Aortic (valve) insufficiency |
| Cyanosis | Cyanoses, unusual change in color of skin to blue, skin cyanosis |

**Table S2. Chain-of-thought (CoT) reasoning prompts used in the error analysis prompting approach. These CoT prompts were derived from the training data of a single fold within the 5-fold cross-validation experiment.**

| *You are given a sentence from a clinical text, if that sentence contains any information related to instances of heart failure, respond with yes and explain why.*  *If not, respond with no and explain why. These words being the signs and evidence:*  *HF, cardiac failure, heart insufficiency, myocardial failure, cardiac insufficiency*  *bilateral leg edema, swelling, dropsy, hydrops, oedema, fluid overload*  *reduced ejection fraction (EF or LVEF), reduced LV function*  *cardiogenic shock, heart shock, cardiovascular collapse, HF exacerbation.*  *If these words are mentioned in the sentence, respond with yes and explain why.*  *If these words are not mentioned in the sentence, respond with no and explain why.*    *Example 1:*  *“The patient has minimal LE edema."*  *Reasoning:*  *Step 1: The phrase “LE edema” directly matches one of the listed indicators: “bilateral leg edema”, which is a known clinical sign of fluid overload.*  *Step 2: Although the sentence does not explicitly mention a diagnosis of heart failure, bilateral edema is a common and recognized symptom associated with congestive heart failure (CHF).*  *Step 3: Since the sentence includes a relevant physical finding from the keyword list, it provides indirect evidence consistent with possible heart failure.*  *Answer:*  *Yes. The sentence mentions “bilateral lower extremities edema,” which matches “bilateral leg edema,” a recognized sign suggestive of heart failure or fluid overload."""*  *Example 2:*  *“General: Sitting on exam table with mild to moderate discomfort Respiratory: Symmetrical chest wall expansion, nonlabored Cardiac: Regular rate and rhythm Integ: No wounds vascular: Nonpalpable distal pulses, palpable bilateral femoral pulses, feet warm and well-perfused, 2+ edema to the right and 1+ edema to the left Assessment and Plan: Testing: Right Lower Limb Ankle Pressure: 131 mm Hg., ABI: 1.04."*  *Reasoning:*  *Step 1: In the vascular section, the sentence notes “2+ edema to the right and 1+ edema to the left”.*  *Step 2: Edema—especially in both lower extremities—is one of the listed indicators: “bilateral leg edema,” “swelling,” “edema,” or “fluid overload”, which can suggest underlying heart failure, particularly when symmetric and without another clear local cause.*  *Step 3: While no explicit diagnosis of heart failure is made in the sentence, the presence of bilateral leg edema is considered a clinical sign consistent with fluid overload, which supports a potential heart failure diagnosis.*  *Answer:*  *Yes. The sentence includes “2+ edema to the right and 1+ edema to the left,” indicating bilateral leg edema, which is a recognized sign of possible heart failure or fluid overload.*  *Example 3:*  *“Localized edema I have advised her that she can increase her morning Lasix pill from 40 up to 60 mg daily for one week, then she is to drop it back down to 40 mg."*  *Reasoning:*  *Step 1: The sentence mentions “localized edema”, which falls under the broader category of “edema”, a listed clinical sign of potential heart failure or fluid overload.*  *The sentence also references Lasix (furosemide), a loop diuretic commonly used to manage volume overload in heart failure patients.*  *Step 2: The instruction to increase the dose of Lasix in response to edema suggests that the clinician is treating fluid retention, which is strongly associated with heart failure management.*  *Answer:*  *Yes. The sentence discusses “localized edema” and increasing Lasix dosage, which suggests treatment of fluid overload—commonly seen in heart failure—thus indicating possible underlying heart failure.*  *Example 4:*  *“Cardiovascular: Positive for leg swelling."*  *Reasoning:*  *Step 1: The sentence mentions “leg swelling”.*  *Step 2: “leg swelling” is one of the listed signs and evidence related to heart failure.*  *Answer:*  *Yes. The sentence mentions leg swelling , which is a recognized sign and can be associated with heart failure."""* |
| --- |
